# Correction: Enhanced catalytic activity of solubilised species obtained by counter-cation exchange of K{Co^II^_1.5_[Fe^II^(CN)_6_]} for water oxidation

**DOI:** 10.1039/d4sc90243b

**Published:** 2024-12-18

**Authors:** Yusuke Seki, Takashi Nakazono, Hiroyasu Tabe, Yusuke Yamada

**Affiliations:** a Chemistry and Bioengineering, Graduate School of Engineering, Osaka Metropolitan University Sugimoto, Sumiyoshi-ku Osaka 558-8585 Japan ymd@omu.ac.jp; b Research Center for Artificial Photosynthesis, Osaka Metropolitan University Sugimoto, Sumiyoshi-ku Osaka 558-8585 Japan; c Institute for Integrated Cell-Material Sciences, Institute for Advanced Study, Kyoto University Yoshida-Honmachi, Sakyo-ku Kyoto 606-8501 Japan

## Abstract

Correction for ‘Enhanced catalytic activity of solubilised species obtained by counter-cation exchange of K{Co^II^_1.5_[Fe^II^(CN)_6_]} for water oxidation’ by Yusuke Seki *et al.*, *Chem. Sci.*, 2024, **15**, 16760–16767, https://doi.org/10.1039/D4SC04390A.

The authors regret that the ESI-MS data shown in Fig. 5, S16 and S17† were not correct. The multiple peaks that appeared in the range of *m*/*z* = 800–2300 were derived from contaminated dimethylpolyiloxane, (Si(CH_3_)_2_O)_*n*_, used as a lubricant of a syringe. ESI-MS measurements carried out with extreme care to avoid this contamination manifested that decanuclear clusters, {Co_6_[Fe(CN)_6_]_4_}^*n*−^, not heptanuclear clusters, {Co_4_[Fe(CN)_6_]_3_}^*n*−^, were formed by the depolymerization, as indicated in the new Fig. 5. Based on this result, the words “heptanuclear clusters of {Co_4_[Fe(CN)_6_]_3_}^*n*−^” and “{Co_4_[Fe(CN)_6_]_3_}^*n*−^” in the original manuscript should be replaced with “decanuclear clusters of {Co_6_[Fe(CN)_6_]_4_}^4−^” and “{Co_6_[Fe(CN)_6_]_4_}^*n*−^”, respectively, and the chemical structures in Fig. 6 and the TOC graphic need correction. The figures, words and phrases based on the correct ESI-MS are indicated below.
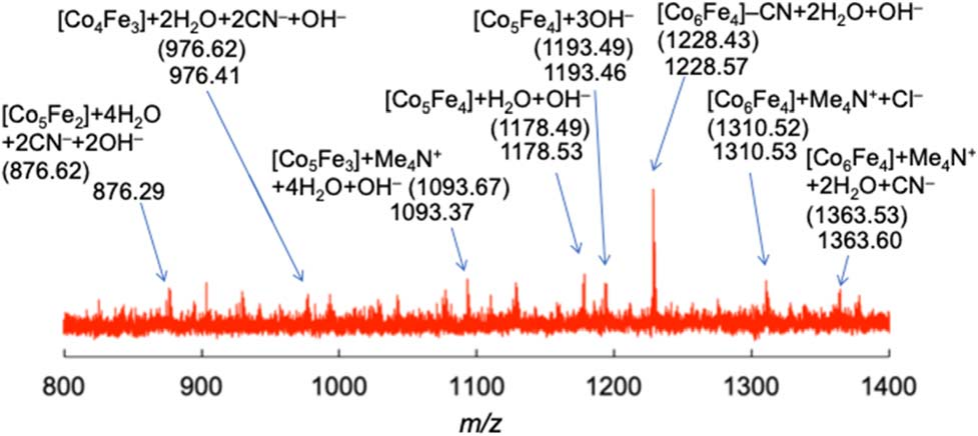


**Fig. 5** Electrospray ionization mass spectrum (negative) of (Me_4_N){Co^II^[Fe^II^(CN)_6_]} {(Me_4_N)**Co–Fe**} in a mixed solvent of water and acetonitrile [1 : 39 (v/v)]. The numbers in parentheses are calculated mass numbers for each species. {Co_*x*_[Fe(CN)_6_]_*y*_}^*n*−^ species are indicated as [Co_*x*_Fe_*y*_].
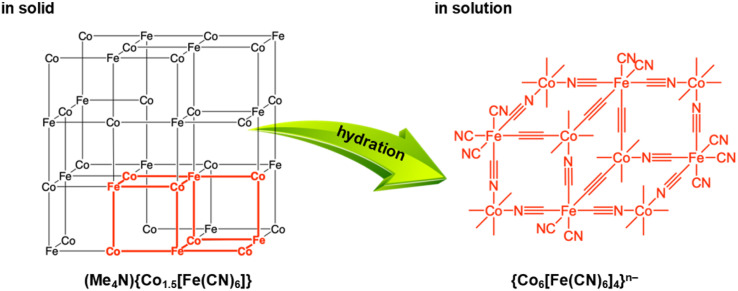


**Fig. 6** Depolymerisation by hydration of frameworks of (Me_4_N){Co^II^_1.5_[Fe^II^(CN)_6_]} {(Me_4_N)**Co–Fe**} with many vacancies into {Co_6_[Fe(CN)_6_]_4_}^*n*−^.

TOC graphic:
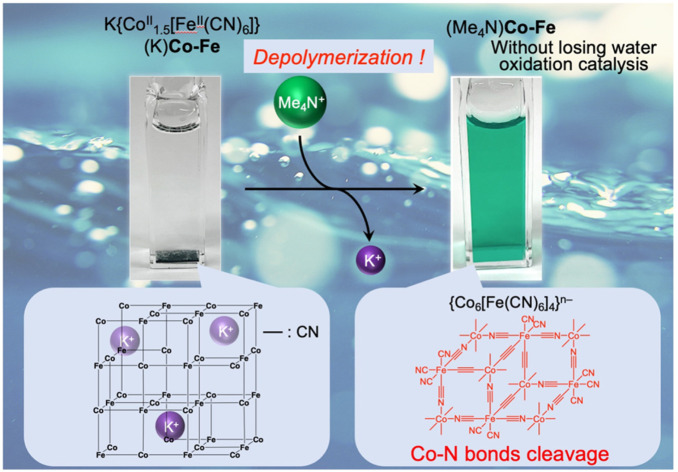


Summary of original and corrected words and phrases in the main text:

**Table:** 

Page, Line	Original	Correct
P. 16760, L. 8 (abstract)	Heptanuclear cluster of {Co_4_[Fe(CN)_6_]_3_}^4−^	Decanuclear clusters of {Co_6_[Fe(CN)_6_]_4_}^4−^
P. 16765, L. 15 (left)	17 anion peaks, which appeared equally spaced with the width of *m*/*z* = 74 derived from Me_4_N^+^ ions (Fig. 5 and S16†)	Many anion peaks
P. 16765, L. 17 (left)	Each peak was composed of {Co_4_[Fe(CN)_6_]_3_}^*n*−^, Me_4_N^+^, and extra-ligands, such as H_2_O, OH^−^, or Cl^−^	Each peak was composed of {Co_*x*_[Fe(CN)_6_]_*y*_}^*n*−^ (*x* = 4–6; *y* = 2–4; [Co_*x*_Fe_*y*_]), Me_4_N^+^, and extra-ligands, such as H_2_O, OH^−^, or Cl^−^(Fig. 5 and S16†)
P. 16765, L. 19 (left)	For example, the observed pattern containing five Me_4_N^+^ ions around *m*/*z* = 1312.17 agreed with the simulated pattern calculated under the assumption that {Co_4_[Fe(CN)_6_]_3_}^*n*−^ with [4 × OH^−^], [H_2_O + 3 × OH^−^], [2 × Cl^−^], and [2 × H_2_O + Cl^−^] is concomitantly generated (Fig. S16†)	For example, the observed pattern around *m*/*z* = 1310.53 agreed with the simulated pattern calculated under the assumption that {Co_6_[Fe(CN)_6_]_4_}^*n*−^ with [Me_4_N^+^ and Cl^−^] and [Me_4_N^+^ and 2H_2_O] is concomitantly generated (Fig. S16†)
P. 16765, L. 35 (left)	{Co_4_[Fe(CN)_6_]_3_}^4−^	{Co_6_[Fe(CN)_6_]_4_}^4−^
P. 16765, L. 11 (right)	Heptanuclear cluster of {Co_4_[Fe(CN)_6_]_3_}^4−^	Decanuclear clusters of {Co_6_[Fe(CN)_6_]_4_}^4−^
P. 16765, L. 25 (right)	{Co_4_[Fe(CN)_6_]_3_}^4−^	{Co_6_[Fe(CN)_6_]_4_}^4−^

In spite of the error, the article's conclusions remain valid, because catalysis and solubilization of (Me_4_N){Co^II^_1.5_[Fe^II^(CN)_6_]} {(Me_4_N)**Co–Fe**} are the main topics of this manuscript. We sincerely apologize for this error and any confusion it may have caused.

The Royal Society of Chemistry apologises for these errors and any consequent inconvenience to authors and readers.

## Supplementary Material

SC-016-D4SC90243B-s001

