# Supplementary material for: Correction: Enhanced catalytic activity of solubilised species obtained by counter-cation exchange of K{CoII1.5[FeII(CN)6]} for water oxidation
Source: Chem Sci. 2024 Dec 18;16(3):1465–6. doi: 10.1039/d4sc90243b (PMC11654750; doi:10.1039/d4sc90243b)
Supplement: SC-016-D4SC90243B-s001 [file SC-016-D4SC90243B-s001.pdf]

Supplementary Information (SI) for Chemical Science.
This journal is © The Royal Society of Chemistry 2024

This version published 18/12/2024 replaces the previous version published 23/09/2024. Figure S16 and S17 are replaced with a corrected version.

Electronic supplementary information (ESI)

Enhanced catalytic activity of solubilised species obtained by counter-cation exchange of K{$Co^{II}_{1.5}$[$Fe^{II}(CN)_6$]} for water oxidation

Yusuke Seki,[a] Takashi Nakazono,[b] Hiroyasu Tabe,[c] and Yusuke Yamada*[ab]

[a]Department of Chemistry and Bioengineering, Graduate School of Engineering, Osaka Metropolitan University, Sugimoto, Sumiyoshi, Osaka, 558-8585, Japan.

[b]Research Center for Artificial Photosynthesis, Osaka Metropolitan University, Sumiyoshi, Osaka 558-8585, Japan.

[c]Institute for Integrated Cell-Material Sciences, Institute for Advanced Study, Kyoto University, Yoshida-Honmachi, Sakyo-ku, Kyoto 606-8501, Japan.

*E-mail: ymd@omu.ac.jp

Supporting table

Table S1. Chemical compositions of $(Me_4N)\{Co^{II}_{1.5}[Fe^{II}(CN)_6]\}$ {(Me_4N)**Co–Fe**} and $K\{Co^{II}_{1.5}[Fe^{II}(CN)_6]\}$ {(K)**Co–Fe**} determined by X-ray fluorescence (XRF) measurements.

Cyano-bridged coordination polymer	Observed Molar Ratio		
	K/Fe	Co/Fe	Cl/Fe
(Me_4N)**Co–Fe**	0.05	1.4	0.07
(K)**Co–Fe**	0.98	1.4	–

Supporting figures

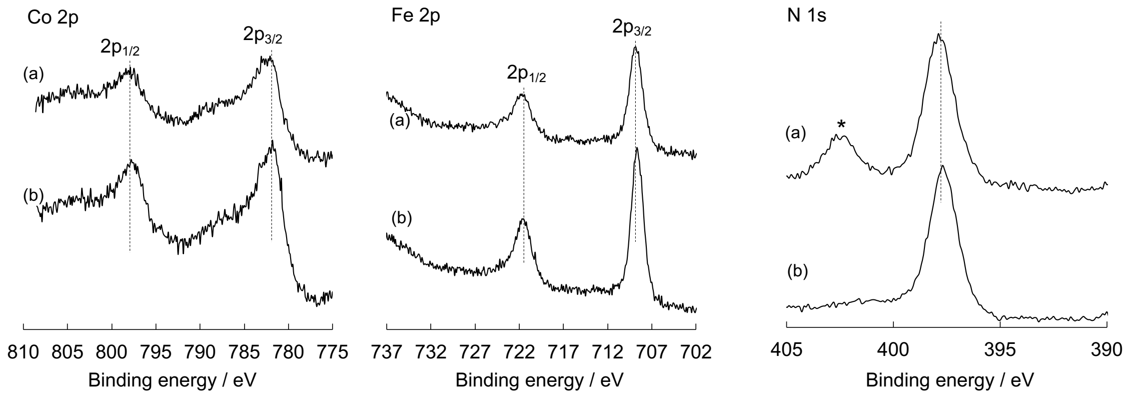

Fig. S1 X-ray photoelectron spectra of (a) $(Me_4N)\{Co^{II}_{1.5}[Fe^{II}(CN)_6]\}$ {(Me_4N)**Co–Fe**} and (b) $K\{Co^{II}_{1.5}[Fe^{II}(CN)_6]\}$ {(K)**Co–Fe**} in Co 2p, Fe 2p, and N 1s regions. *Attributed to Me_4N^+ ion.

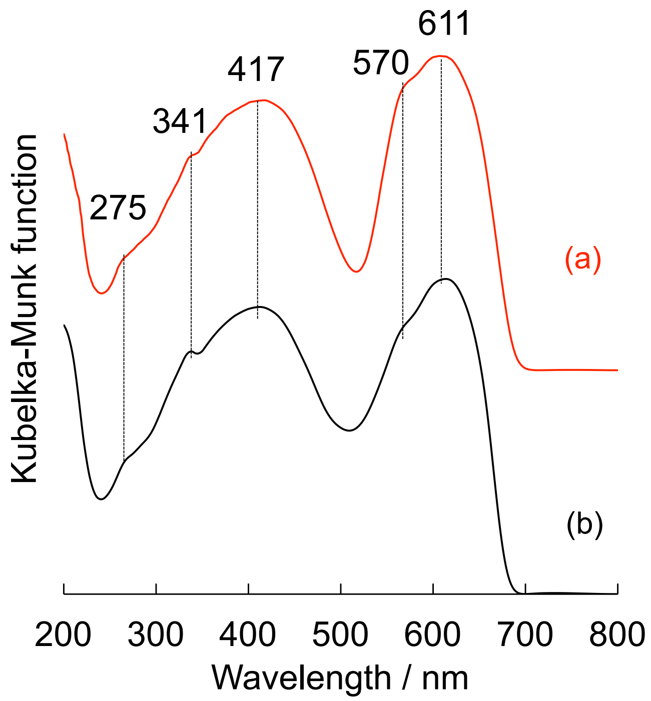

Fig. S2 Diffused reflectance UV-vis spectra of (a) $(Me_4N)\{Co^{II}_{1.5}[Fe^{II}(CN)_6]\}$ {(Me_4N)**Co–Fe**} and (b) $K\{Co^{II}_{1.5}[Fe^{II}(CN)_6]\}$ {(K)**Co–Fe**}. The numbers indicate the wavelengths of absorption maxima.

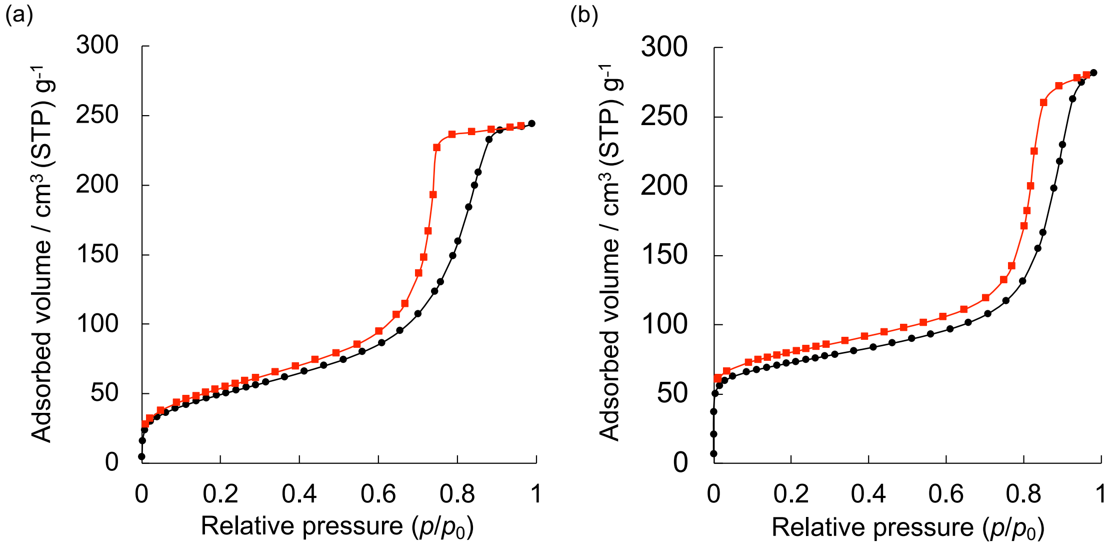

Fig. S3 Nitrogen adsorption (black circles)-desorption (red squares) isotherms of (a) $(Me_4N)\{Co^{II}_{1.5}[Fe^{II}(CN)_6]\}$ {(Me_4N)**Co–Fe**} and (b) $K\{Co^{II}_{1.5}[Fe^{II}(CN)_6]\}$ {(K)**Co–Fe**} at –196°C.

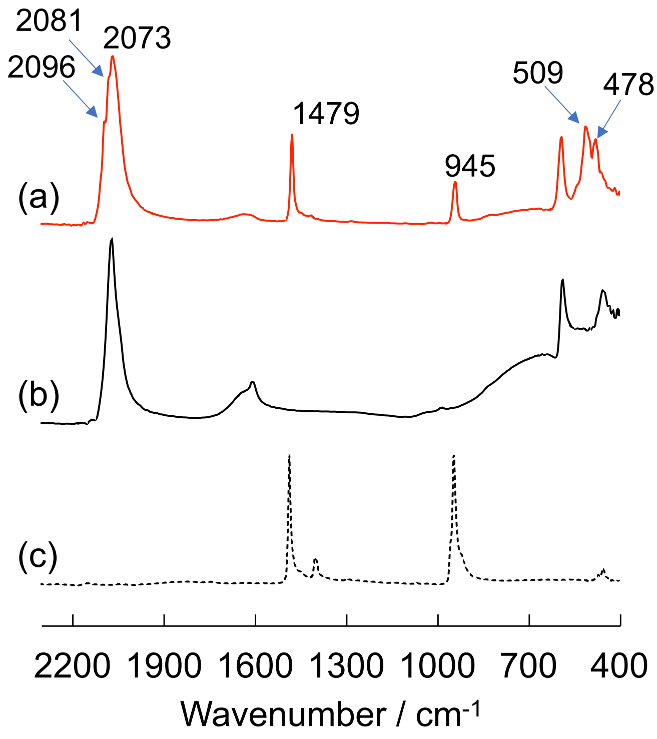

Fig. S4 Infrared (IR) spectra of (a) $(Me_4N)\{Co^{II}_{1.5}[Fe^{II}(CN)_6]\}$ {(Me_4N)**Co–Fe**}, (b) $K\{Co^{II}_{1.5}[Fe^{II}(CN)_6]\}$ {(K)**Co–Fe**}, and (c) Me_4NCl. The spectra were obtained by the IR spectrometer with a diamond ATR attachment.

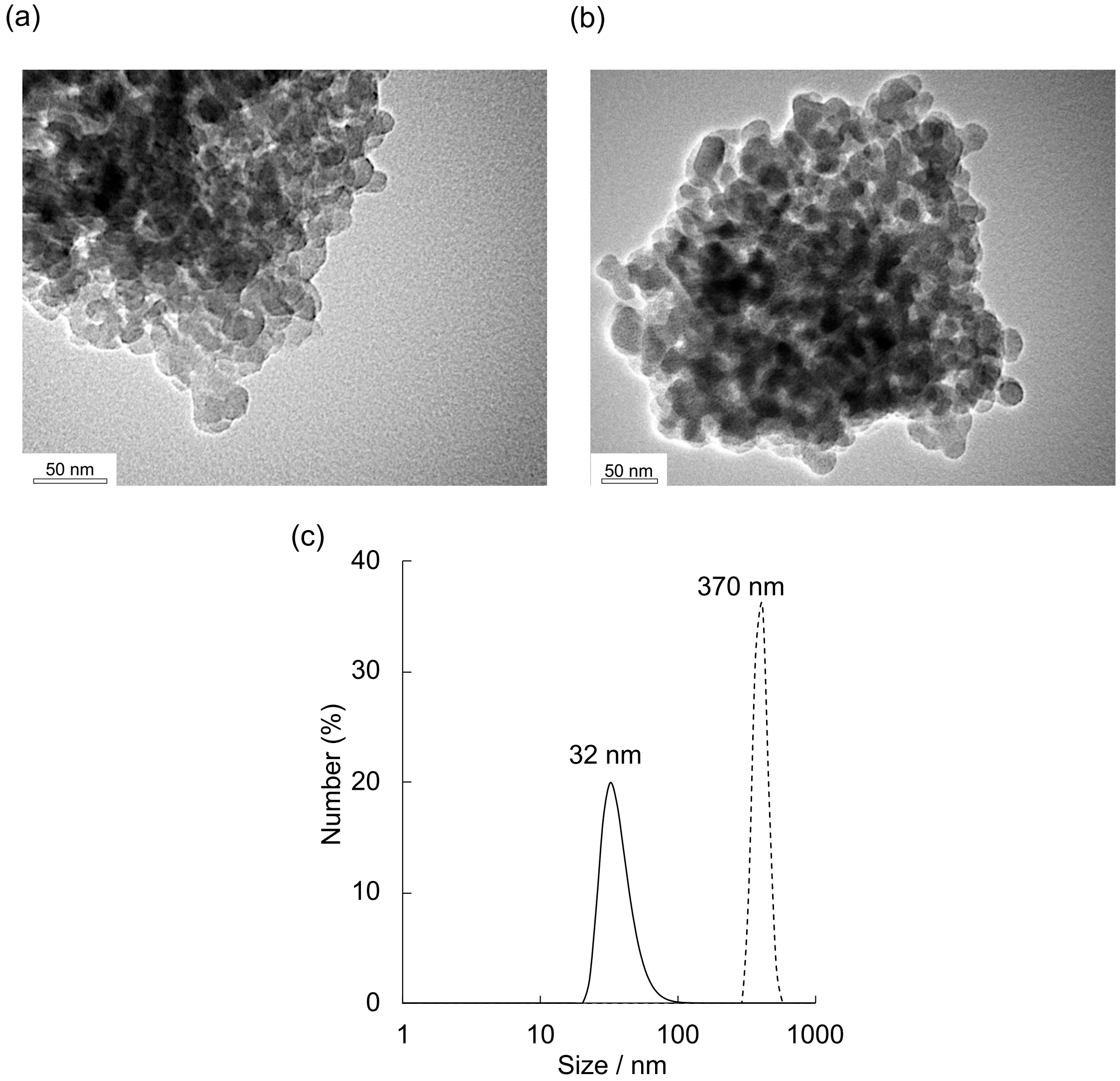

Fig. S5 (a) Transmission electron microscope (TEM) images of (a) $(Me_4N)\{Co^{II}_{1.5}[Fe^{II}(CN)_6]\}$ $\{(Me_4N)$**Co–Fe**$\}$ and (b) $K\{Co^{II}_{1.5}[Fe^{II}(CN)_6]\}$ $\{(K)$**Co–Fe**$\}$. (c) Size distribution of (Me_4N)**Co–Fe** (solid line) and (K)**Co–Fe** (dotted line) determined by dynamic light scattering (DLS).

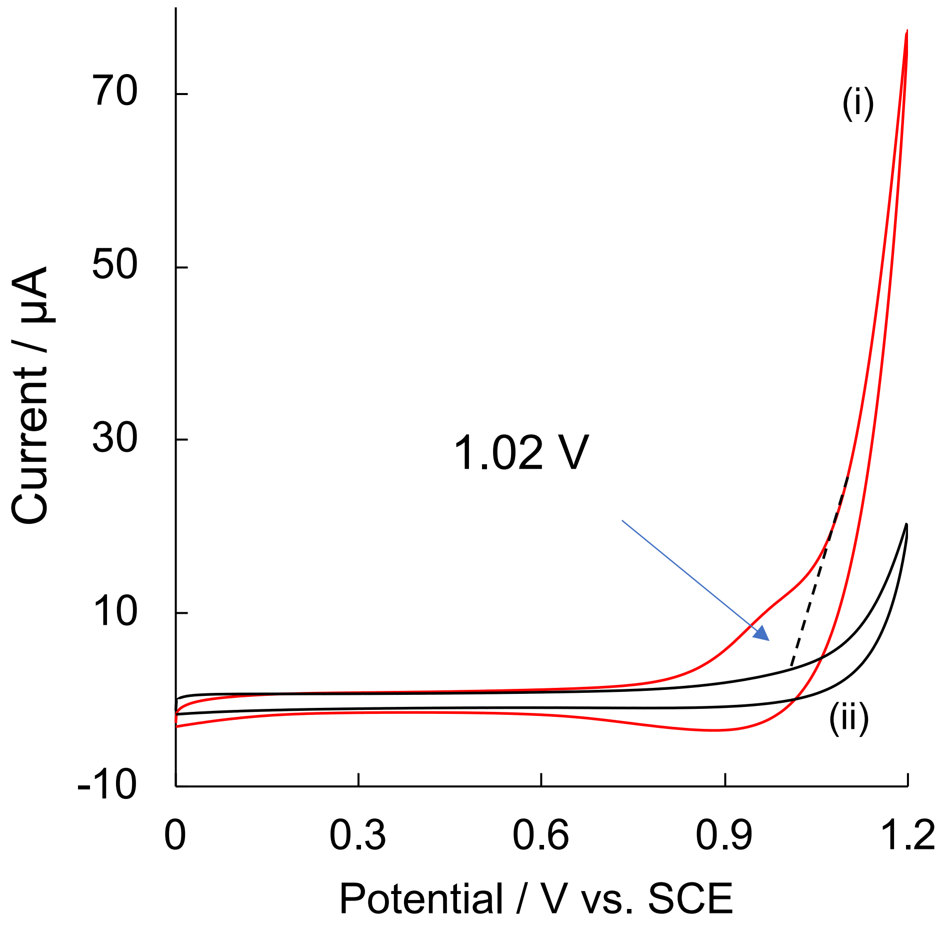

Fig. S6 Cyclic voltammetry of (i) $(Me_4N)\{Co^{II}_{1.5}[Fe^{III}(CN)_6]\}$ {(Me_4N)**Co–Fe**, 1.0 mM_{Fe}} and (ii) blank in a phosphate buffer (pH 7.0, 50 mM, 3.0 mL) recorded at the sweep rate of 100 mV s^{-1}. The working, counter, and reference electrodes were a glassy carbon, a Pt wire, and SCE, respectively.

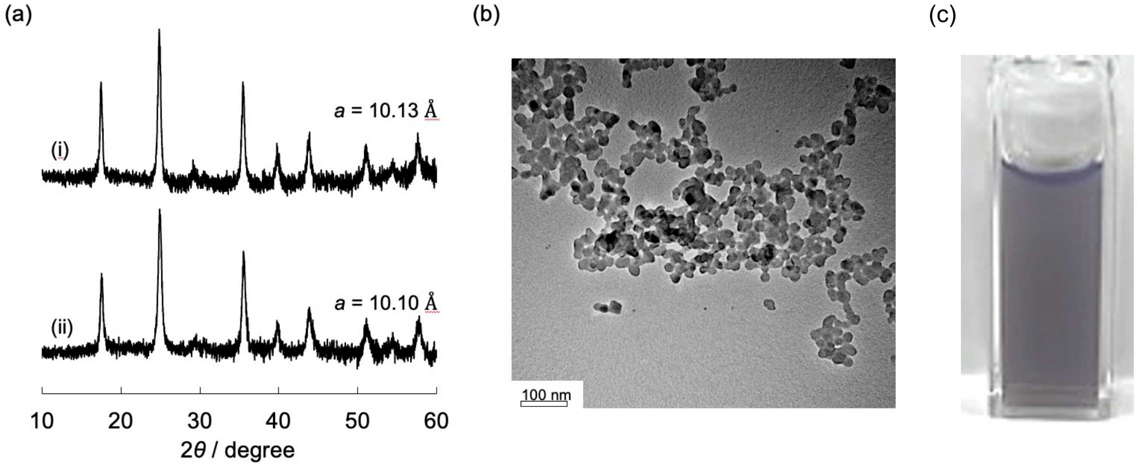

Fig. S7 (a) Powder X-ray diffraction (PXRD) patterns of (i) K{$Co^{II}_{1.5}$[$Fe^{II}(CN)_6$]} {(K)**Co–Fe**} and (ii) water-dispersible (K)**Co–Fe** (modified with [$Fe^{II}(CN)_6$]$^{4-}$). (b) TEM image of water-dispersible (K)**Co–Fe**. (c) water-dispersible (K)**Co–Fe** (modified with [$Fe^{II}(CN)_6$]$^{4-}$) in water.

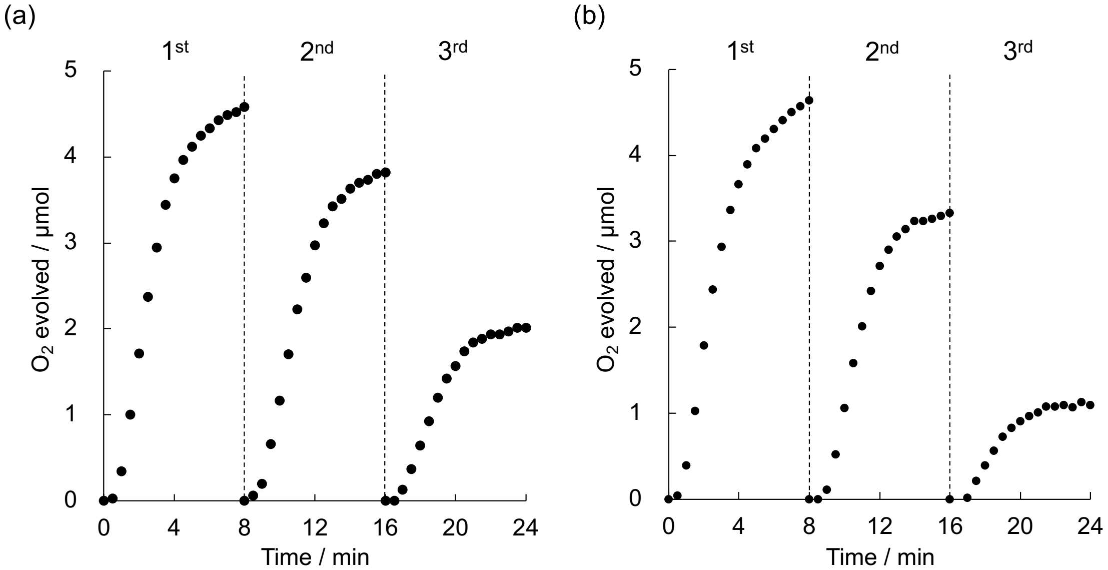

Fig. S8 Repetitive experiments of photocatalytic water oxidation under photoirradiation of a phosphate buffer (pH 8.0, 50 mM, 2.0 mL) containing $Na_2S_2O_8$ (5.0 mM, 10 µmol), [$Ru(bpy)_3$]SO_4 (0.30 mM, 0.6 µmol) and (Me_4N){$Co^{II}_{1.5}$[$Fe^{II}(CN)_6$]} {(Me_4N)**Co–Fe**}, 80 μM_{Fe}} with an LED light. A small aliquot (100 µL) of (a) $Na_2S_2O_8$ (10 µmol) and [$Ru(bpy)_3$]SO_4 (0.6 µmol) and (b) $Na_2S_2O_8$ (10 µmol) was added to the reaction solution after each run.

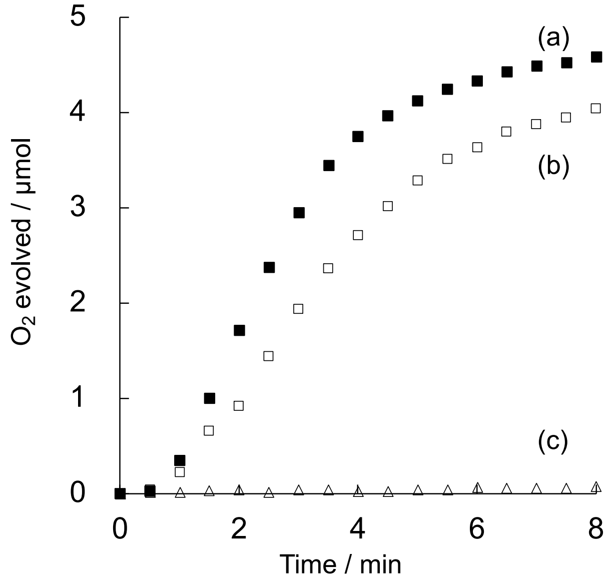

Fig. S9 Time courses of O_2 evolution under visible-light irradiation of a phosphate buffer (pH 8.0, 50 mM, 2 mL) containing $Na_2S_2O_8$ (5.0 mM), $[Ru(bpy)_3]SO_4$ (0.30 mM) and a catalyst (80 μM_{Fe}): (a) $(Me_4N)\{Co^{II}_{1.5}[Fe^{II}(CN)_6]\}$ {(Me_4N)**Co–Fe**, closed square}, (b) $Co^{II}(NO_3)_2$ (open square), or (c) $K_4[Fe^{II}(CN)_6]$ (open triangle) with LED light.

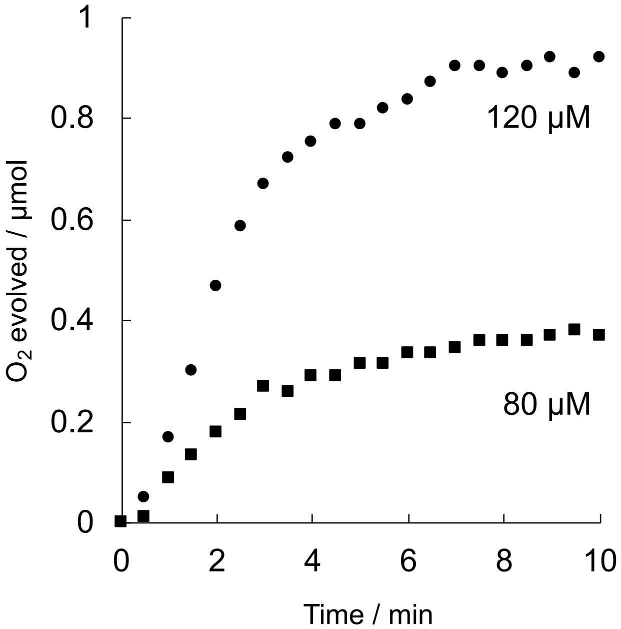

Fig. S10 Thermal water oxidation using $(Me_4N)\{Co^{II}_{1.5}[Fe^{II}(CN)_6]\}$ {(Me_4N)**Co–Fe**, 120 μM_{Fe}} and $[Ru^{III}(bpy)_3]^{3+}$ (4 µmol) in a phosphate buffer (pH 8.0, 50 mM) and H_2SO_4 aq. (50 mM) (19:1 (v/v), 2.0 mL).

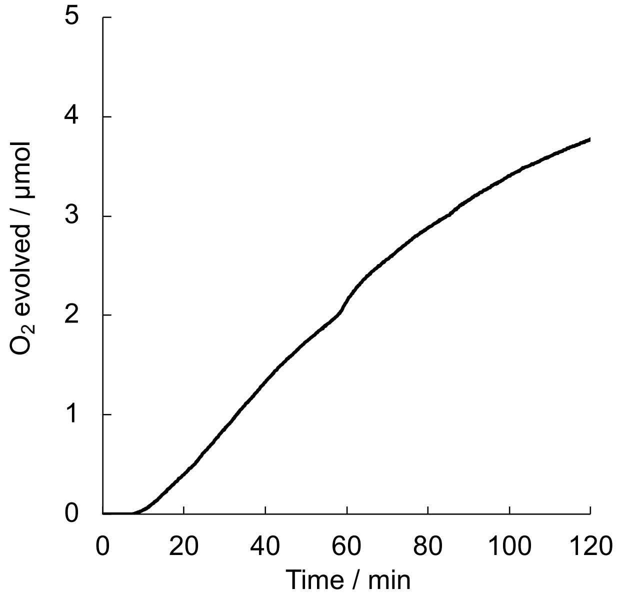

Fig. S11 Time course of O_2 evolution under visible-light irradiation (λ = 450 nm) of a phosphate buffer (pH 8.0, 50 mM, 2 mL) containing $Na_2S_2O_8$ (5.0 mM), $[Ru(bpy)_3]SO_4$ (0.30 mM) and $(Me_4N)\{Co^{II}_{1.5}[Fe^{II}(CN)_6]\}$ {(Me_4N)**Co–Fe**, 80 μM_{Fe}} in a quartz cuvette (light path length: 1.0 cm).

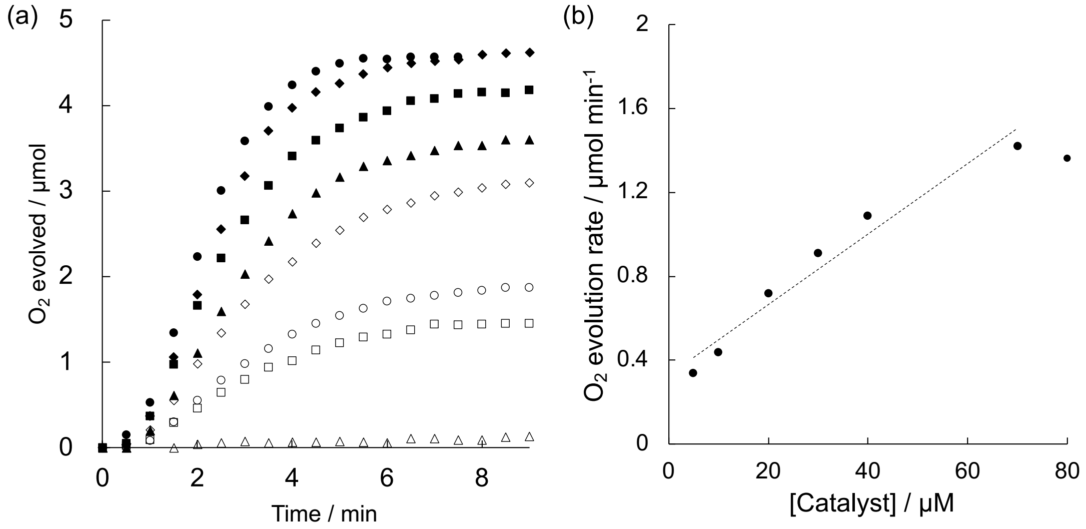

Fig. S12 (a) Time courses of O_2 evolution under visible-light irradiation of a phosphate buffer (pH 8.0, 50 mM, 2.0 mL) containing $Na_2S_2O_8$ (5.0 mM), $[Ru(bpy)_3]SO_4$ (0.30 mM) and $(Me_4N)\{Co^{II}_{1.5}[Fe^{II}(CN)_6]\}$ {(Me_4N)**Co–Fe**} (80 μM_{Fe}; closed diamond, 70 μM_{Fe}; closed circle, 40 μM_{Fe}; closed square, 30 μM_{Fe}; closed triangle, 20 μM_{Fe}; closed diamond, 10 μM_{Fe}; open circle, 5 μM_{Fe}; open square, no catalyst; open triangle) with LED light. (b) Plots of initial O_2-evolution rates at various concentrations of (Me_4N)**Co–Fe**. The initial rates were calculated by the slopes of the plots from 1 to 3.5 min.

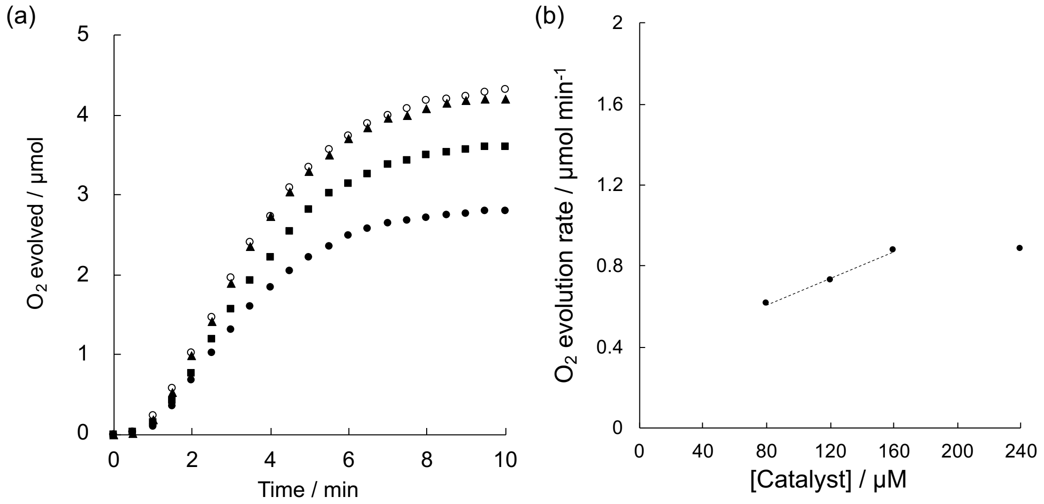

Fig. S13 (a) Time courses of O_2 evolution under visible-light irradiation of a phosphate buffer (pH 8.0, 50 mM, 2.0 mL) containing $Na_2S_2O_8$ (5.0 mM), $[Ru(bpy)_3]SO_4$ (0.30 mM) and $K\{Co^{II}_{1.5}[Fe^{II}(CN)_6]\}$ {(K)**Co–Fe**: 80 μM_{Fe}, closed circle; 120 μM_{Fe}, closed square; 160 μM_{Fe}, closed triangle; 240 μM_{Fe}, open circle} with LED light. (b) Initial O_2-evolution rates depending on the concentrations of (K)**Co–Fe**. The initial rates were calculated from the slopes of the plots in (a) from 1 to 3.5 min.

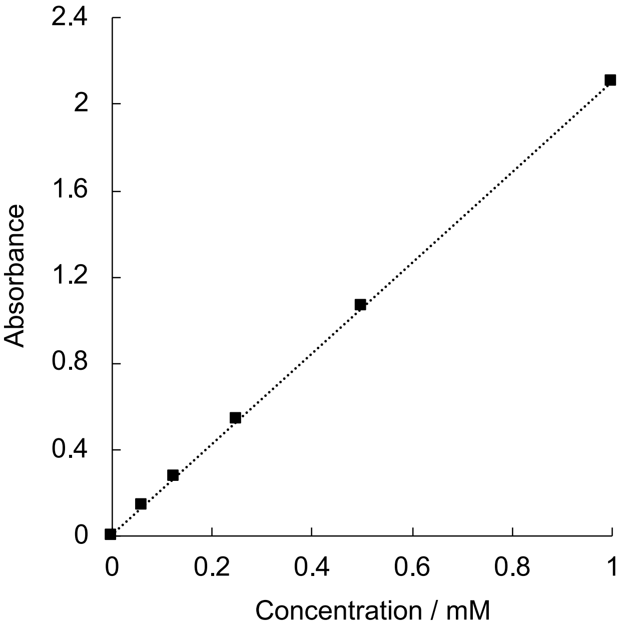

Fig. S14 Absorbance at 615 nm as a function of the concentrations of (Me_4N)**Co–Fe** in the mixed solution of water and acetonitrile [1:39 (v/v)].

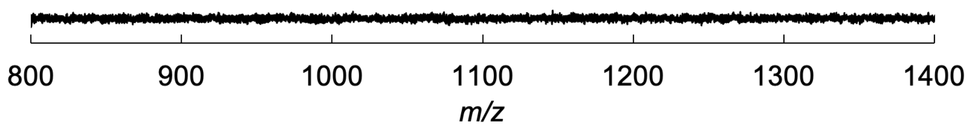

Fig. S15 Electrospray ionization mass spectrum (ESI-MS) measured for a mixed solution of water and acetonitrile [1:39 (v/v)] without solute (background).

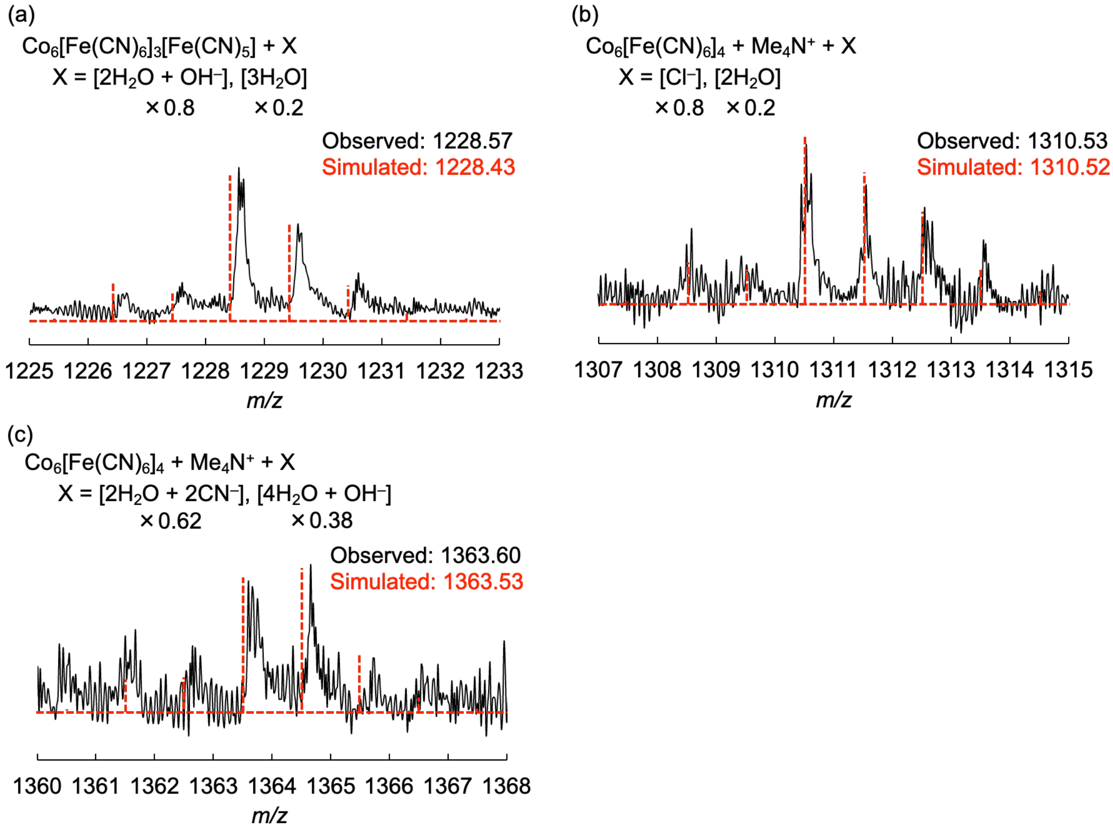

Fig. S16 Isotope patterns (black solid lines) of ESI-MS peaks (negative mode) appeared around (a) 1228.58, (b) 1310.53, and (c) 1363.60 at the measurements of a mixed solution of water and acetonitrile [1:39 (v/v)] containing $(Me_4N)\{Co^{II}_{1.5}[Fe^{II}(CN)_6]\}$ {(Me4N)**Co–Fe**}. The simulated patterns (red dotted lines) were calculated under the assumption that a couple of species containing decanuclear metal complex, Me_4N^+, and extra-ligands such as H_2O, OH^-, CN^-, and Cl^- ions were concomitantly generated.

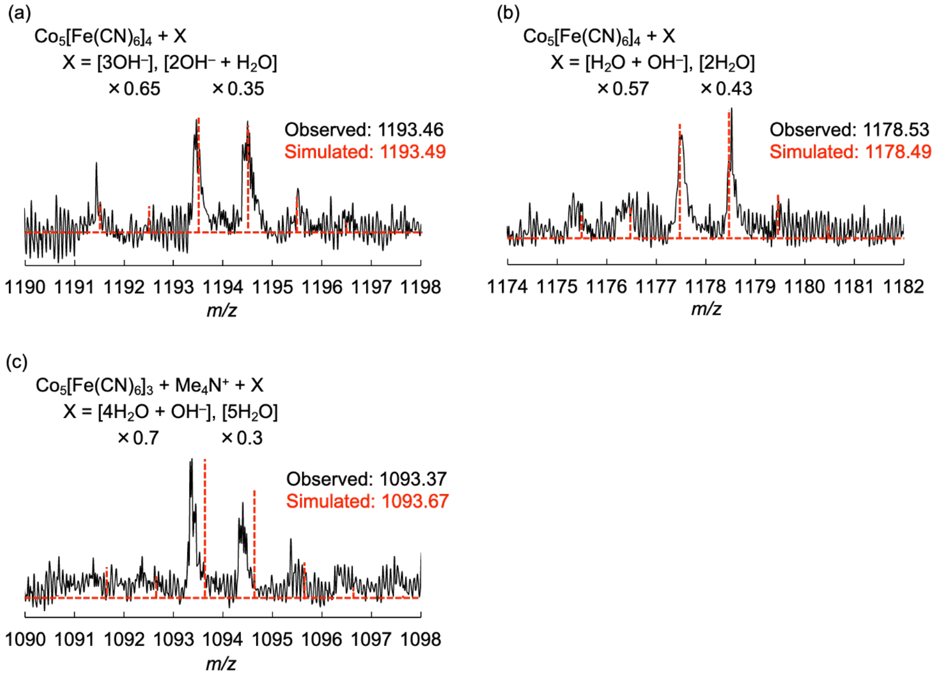

Fig. S17 Isotope patterns (black solid lines) of ESI-MS peaks (negative mode) appeared around (a) 1193.46, (b) 1178.53, and (c) 1093.37 at the measurements of a mixed solution of water and acetonitrile [1:39 (v/v)] containing $(Me_4N)\{Co^{II}_{1.5}[Fe^{II}(CN)_6]\}$ {(Me_4N)**Co–Fe**}. The simulated patterns (red dotted lines) were calculated under the assumption that a couple of species containing $\{Co_5[Fe(CN)_6]_4\}^{n-}$ or $\{Co_5[Fe(CN)_6]_3\}^{n-}$ derived from $\{Co_6[Fe(CN)_6]_4\}^{n-}$, Me_4N^+, and extra-ligands such as H_2O and OH^-, were concomitantly generated.

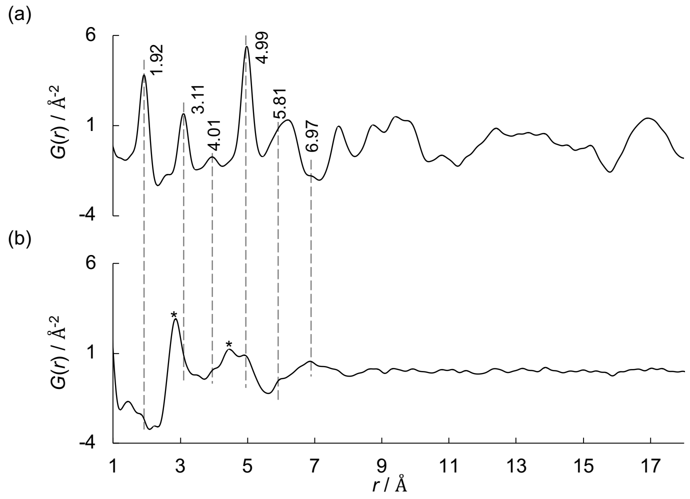

Fig. S18 Pair distribution function (PDF) of (a) $(Me_4N)\{Co^{II}_{1.5}[Fe^{II}(CN)_6]\}$ {(Me_4N)**Co–Fe**} and (b) (Me_4N)**Co–Fe** in water. *Attributed to water.[S1]

References

S1 A. H. Narten and W. E. Thiessen, L. Blum, *Science* 1982, **217**, 1033.
